# Hajdu Cheney Syndrome due to NOTCH2 defect – First case report from Pakistan and review of literature

**DOI:** 10.1016/j.amsu.2021.01.041

**Published:** 2021-01-19

**Authors:** Sibtain Ahmed, Aahan Arif, Saadia Abbas, Muhammad Osama Khan, Salman Kirmani, Aysha Habib Khan

**Affiliations:** a Section of Clinical Chemistry, Department of Pathology and Laboratory Medicine, Aga Khan University, Stadium Road, P.O. Box 3500, Karachi, 74800, Pakistan; b Medical College, Aga Khan University. Stadium Road, P.O. Box 3500, Karachi, 74800, Pakistan; c Department of Paediatrics & Child Health, Aga Khan University. Stadium Road, P.O. Box 3500, Karachi, 74800, Pakistan

**Keywords:** Hajdu cheney syndrome, Case report, *NOTCH2,* Pakistan

## Abstract

**Introduction and importance:**

Hajdu Cheney Syndrome (HCS) is a rare skeletal disease characterized by severe, progressive focal bone loss with osteoporosis, variable craniofacial, vertebral anomalies and distinctive facial features. It is inherited as an autosomal dominant disease although sporadic cases have been described in literature. Identifying these cases in clinical practice is important for proper diagnosis and management.

**Case presentation:**

We report a case of a 36-year-old male patient presented at metabolic bone disease clinic at the Aga Khan University Hospital with history of multiple fragility fractures and juvenile osteoporosis since childhood. DNA sequence analysis of the NOTCH2 coding sequence revealed a pathogenic variant in NOTCH 2, Exon 34, c.6426_6427insTT (p.Glu2143Leufs*5), consistent with a NOTCH2 related conditions including HCS.

**Clinical discussion:**

The multitude of presentations associated with HCS are linked to the NOTCH2 gene, as Notch signaling is one of the core signaling pathways that control embryonic development. Hence, mutations in the Notch signaling pathway cause developmental phenotypes that affect various organs including the liver, skeleton, heart, eye, face, kidney, and vasculature.

**Conclusion:**

To the best of our knowledge, nucleotide mutations of c.6933delT, c.6854delA, c.6787C.T, and c.6424_-_6427delTCTG were all determined to be novel, with c.6428T > C being the most common mutation found in literature. The c.6426_6427insTT mutation our patient was found to have via gene sequencing too appears to be a novel mutation, which has not previously been reported in literature.

## Highlights

• Hajdu Cheney Syndrome (HCS) is a rare skeletal disease.
• The multitude of presentations associated with HCS are linked to the NOTCH2 gene.
• We report a case of a 36-year-old male Pakistani patient with multiple fragility fractures and acroosteolysis.

## 1 Introduction

Hajdu Cheney Syndrome (HCS) is a rare skeletal disease characterized by severe, progressive focal bone loss with osteoporosis, variable craniofacial, vertebral anomalies and distinctive facial features. It is inherited as an autosomal dominant disease although sporadic cases have been described in literature [1]. There is marked phenotypic variability and the degree of severity in clinical features between affected individuals and hence it often remains undiagnosed until adolescence or adulthood [2]. Thus, despite the fact that the disease begins to manifest at birth, it is still extremely rare with fewer than one hundred cases found in the literature [3].

The disease was linked to mutations in the Notch Receptor 2 (*NOTCH2*) gene in 2011 by whole-exome sequencing in individuals with HCS. Specifically, mutations of exon 34 have been shown to remove PEST domains, a peptide sequence that is rich in proline (P), glutamic acid (E), serine (S), and threonine (T) due to a premature stop in amino acid codon, creating a truncated and stable NOTCH2 protein with enhanced NOTCH2 signaling activity [1,[4], [5], [6]]. Dysregulation of Notch signaling is associated with skeletal developmental disorders and bone remodeling, and gain-of-function mutations of *NOTCH2* are associated with HCS [3]. Notch receptors are single-pass trans membrane proteins that determine cell fate and play a critical role in skeletal development and homeostasis [3]. Furthermore, NOTCH2 is present in all embryonic tissue and hence manifested as wide phenotype with a variety of clinical manifestations, related to skeletal development & homeostasis [1].

Identifying these cases in clinical practice is important for proper diagnosis and management. The unknown mechanism of disease progression and bone loss amongst affected individuals poses a unique therapeutic challenge. Continued research is critically important to build on prior knowledge so that advancement related to drug development and treatment in the rare diseases of bone to improve bone health can be made.

We present the first case diagnosed with HCS from our metabolic bone disease clinic at Aga Khan University Hospital, Karachi, Pakistan. We also performed a review of the literature to understand the phenotypic and genotypic spectrum of the disease, and treatment regimens utilized for its management. We searched the MEDLINE database for studies with the search terms “*NOTCH2*” and “Hajdu Cheney Syndrome” from 2012 to 2019 with English language restrictions. The title, abstract and full text of all documents identified according to these search criteria were scrutinized by the authors. Additionally, all references found in the published articles were also reviewed for case report ascertainment. This is an attempt to analyze the complete phenotype of HCS for better understanding of the syndrome and contribute to an earlier diagnosis in new cases. Further, it is also important for genetic counselling. Written informed consent was obtained from the patient for publication of this case report. This work has been reported in line with the Case Report (CARE) guidelines [7,8].

## 2 Case report

A 36-year-old male patient presented at metabolic bone disease clinic at the Aga Khan University Hospital with history of multiple fragility fractures and juvenile osteoporosis since childhood. There was no history of neurological or cardiovascular complications nor any urinary symptoms.

Upon physical examination, the patient's height was 159 cm and weight was 95 kg. He displayed a prominent forehead, hypertelorism, long eyelashes, and micrognathia. Furthermore, his hands were short and stubby with acroosteolysis and broad dark nails. There were no signs of local inflammation in either fingers or toes. He had lost all his teeth and had replaced them with dentures. The liver and spleen were not palpable and no abnormalities were noted on cardiovascular and neurological exams. Previous consultations in the United States of America, during his childhood had considered the possibility of HCS due to the presence of acroosteolysis. He had been taking bisphosphonates including Pamidronate and Zoledronic Acid since childhood. He was on Prolia when he presented this time.

Table 1 shows the results of bone biochemistry and Dual-energy X-ray absorptiometry (DXA) findings at an interval of two years. Complete blood count, serum electrolytes, liver and renal function tests (except gamma-GT, which was marginally raised) were within the normal range on different occasions. In addition, thyroid profile, follicle stimulating hormone, and luteinizing hormone & free androgen index were also within the reference intervals.

**Table 1:** Results of bone biochemistry & DXA findings of the patient in 2019 & 2017.

Variable		2019	2017
**Bone Biochemistry**			
Serum Calcium (8.6–10.2mg/dl)		9.3	9.4
Serum Phosphorus (2.5–4.5 mg/dl)		3.3	2.9
Serum Magnesium (1.6–2.6 mg/dl)		1.7	2.0
Serum 25-hydroxy vitamin D (>20ng/ml)		36.1	31.9
Serum 1,25-hydroxy vitamin D (19.9–79.3 pg/mL)		–	46.7
Parathyroid hormone (PTH) (16–87pg/ml)		59.9	55.5
Serum N-terminal telopeptide (NTx) (5.4–24.2 nMBCE/L)		13.93	16.69
**DXA Findings**			
Left Hip	BMD	0.863	0.866
	Z score	−1.0	−1.1
Femoral Neck (FN)	BMD	0.646	0.666
	Z score	−1.7	−1.6
Spine	BMD	−4.4	0.598
	Z score	−4.3	−4.5
L (distal) forearm	BMD	0.617	0.649
	Z score	−3.6	−2.9

Based on history & facial dysmorphism, he was referred for geneticist consult. DNA sequence analysis of the NOTCH2 coding sequence was performed on genomic DNA extracted from peripheral leucocytes at the Invitae Corporation, San Francisco CA 94103. A pathogenic variant in NOTCH2, Exon 34, c.6426_6427insTT (p.Glu2143Leufs*5) heterozygous was identified, consistent with a predisposition to, or diagnosis of NOTCH2 related conditions including HCS (MedGen UID: 182961) and Alagille syndrome 2 (ALGS2) (MedGen UID: 341844).

Further inquiry revealed that the patient's daughter has facial features resembling those of her father and a history of patent ductus arteriosus (PDA), acroosteolysis. He also gave history of abortion of a female fetus with cardiac developmental defect. Genetic testing was advised for confirmation of the disease and subsequent management.

During his most recent follow-up visit, the patient was found to be doing well. He was on Prolia and was advised weight reduction, lifestyle measures, and exercise with stretch bands.

## 3 Discussion

HCS was first reported by Hajdu and Kautze in 1948 [7] and later by William D. Cheney in 1965 [[9], [10]]. Prior to the molecular etiology of HCS being discovered, Brennan et al. described 10 key clinical manifestations of HCS including acroosteolysis, Wormian bones, Platybasia, premature loss of teeth, micrognathia, coarse face, coarse hair, midface flattening, short stature (<5%), and a positive family history [11]. He also proposed a diagnostic tool on the basis of a series of physiological parameters and genetic inheritance [11].

**Fig. 1:**
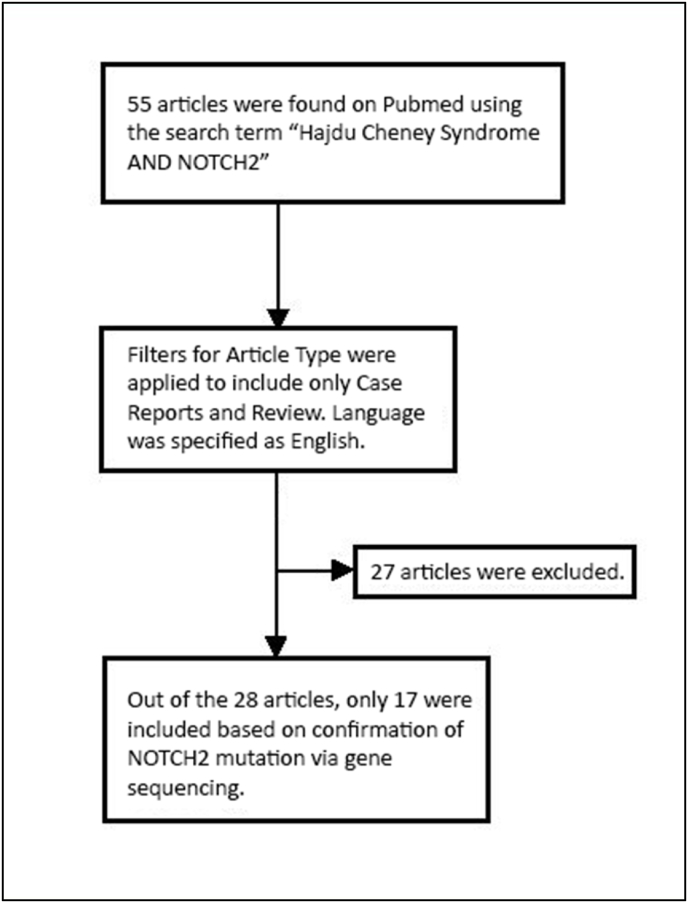
Consort showing the Selection of Manuscripts for Review on Hajdu Cheney Syndrome.

The search strategy adopted for the literature review to understand the phenotype and genotypic variability in patients with confirmed NOTCH2 mutations is summarized in Fig. 1. Table 2, Table 3 shows the full spectrum of phenotypic and radiological manifestations reported so far with confirmed NOTCH2 mutations. Substantial phenotypic variability amongst patients’ results in a syndrome that often remains undiagnosed until adolescence or adulthood despite being congenital and manifesting at birth. Furthermore, the plethora of clinical symptoms leads to many potential differentials amongst physicians who are unaware of this syndrome.

**Table 2:** Phenotypic findings by organ systems & frequency (n) in hajdu cheney syndrome with NOTCH2 mutations reported in literature from 2012 to 2019.

Systems	Manifestation	Frequency (n)
**Craniofacial**	Auricular Abnormalities	21
	Retro/micrognathia	20
	Coarse hair	14
	Long philtrum, Down slanted palpebral fissures	13 cases each
	Hypertelorism	12
	Synophrys	11
	Bushy Eyebrows, Mid facial Hypoplasia, Thin Lips	10 cases each
	Wide nose, Cleft palate	9
	Large head circumference	5
	Prominent forehead	3
	Down turned mouth, Telecanthus	2 cases each
	Short nose, Pale skin, Small face	1 case each
**Dentition**	Loss of teeth	11
	Malocclusion	4
	Irregular wide-spaced teeth & Dental hypermobility	2 cases each
	Short roots of permanent teeth	1
**Cardiovascular**	PDA	7
	Sub aortic stenosis	2
	VSD, Coarctation of the aorta, Hypoplastic left ventricle, Supraventricular tachycardia	1 case each
**Eye**	Exophthalmos	4
	Glaucoma, Myopia	1 case each
**Pulmonary**	Pneumonia, Upper airway obstruction	2 cases each
	Pulmonary hypertension, Idiopathic pulmonary hemosiderosis	1 case each
	Fibrosis of lungs	1
**Renal**	Renal cysts	6
	Polycystic kidney disease	1
	End stage renal disease	1
**Other Findings**	Hyploplasia of the nails, clubbed fingers, Cryptorchidism	3 cases each
	GI malrotation, Polyhydramnios, Inguinal hernia	2 cases each
	Splenomegaly, Anterior crossbite, Psoriatic arthritis	1 case each

**Table 3:** Radiographic Findings in Systems & their Frequency (n) in Hajdu Cheney Syndrome with NOTCH2 Mutation Reported in Literature from 2012 to 2019.

Radiological Findings	Manifestation	Frequency (n)
**HEAD**	Wormian Bones	17
	Platybasia/Basilar Invagination	10
	Dilatation of ventricles	6
	Open skull sutures, Bathrocephaly	5 cases each
	Chiari malformation	3
	Thin corpus callosum, Paranasal sinus hypoplasia, Lack of frontal sinus	2 cases each
	Scaphocephalus, Thin superior cerebellar peduncles, Arachnoid cyst posterior fossa with defect in occipital bone, Tympanic bone incompletely formed, Irregular temporomandibular joint	1 case each
**LIMBS**	Acroosteolysis	23
	Fracture of long bones	7
	Brachydactyly	6
	Fibular bowing (serpentine fibulae), Hallus Valgus/Varus	5
	Radial dislocation	3
	Genu Valgus/Varus, Syndactyly, Hip dysplasia, Clubbed foot, Low trauma fractures	2
	Hip protrusion, Loss of normal femoral epiphysis, Abnormal ankle mortise, Accessory naviculae, Mild bowing of tibia, Planovalgus, Pes Valgus, Pes Planus, Lordosis, Broad valgus toes, Pes cavus, Cubitus valgus	1 case each
**SPINE**	Osteoporosis/osteopenia	21
	Vertebral fractures	11
	Scoliosis- kyphosis	10
	Fishbone deformity	4
	Syringomyelia, Sclerosis of anterior vertebral endplates	3 cases each
	Spondylolisthesis, Degenerative disk disease	1

Acroosteolysis and osteoporosis are two most common radiographic findings followed by Wormian bones & Platybasia. Acroosteolysis usually develops after the first few years of life and progresses through adulthood, while severe osteoporosis occasionally occurs with fishbone deformity [12]. Although Acroosteolysis is a hallmark of HCS, it can also be secondary to a variety of other disorders. These may include autoimmune disorders such as Scleroderma, Systemic Lupus Erythematous, Rheumatoid Arthritis, and Raynaud's Disease; or neuropathies such as Diabetes Mellitus [13]. Other causes include trauma, burns, frostbite, toxic steep (due to PVC or ergot poisoning), and infections [13].

The multitude of presentations associated with HCS are linked to the NOTCH2 gene, as Notch signaling is one of the core signaling pathways that control embryonic development [14]. Hence, mutations in the Notch signaling pathway cause developmental phenotypes that affect various organs including the liver, skeleton, heart, eye, face, kidney, and vasculature. Gene sequencing has revealed a wide array of nucleotide mutations and protein alterations associated with HCS (Table 4). These differences and variability may also account for the diverse phenotypic spectrum as seen in literature. To the best of our knowledge, nucleotide mutations of c.6933delT, c.6854delA, c.6787C.T, and c.6424_-_6427delTCTG were all determined to be novel, with c.6428T > C being the most common mutation found in literature. The c.6426_6427insTT mutation our patient was found to have via gene sequencing too appears to be a novel mutation, which has not previously been reported in literature.

**Table 4:** Spectrum of nucleotide mutations & protein alteration in notch 2 gene in patients with hajdu cheney syndrome reported from 2010 to 2019 (N = 32).

S. No.	Author et al. (ref)	Year	Country	No. of patients	Age at (years)	Gender	Nucleotide Mutation	Protein Alteration
1	Stathopoulos, I et al. [18]	2012	Greece	1	31	F	c.6450delT	p.Pro2149Profs5X
2	Lee, G et al. [19]	2013	Korea	1	21	M	c.6443T > G	p.Leu2148
3	Gu, J et al. [20]	2013	China	1	57	F	c.6622C > T	p.Gln2208X
4	Narumi, Y et al. [21]	2013	Japan	6	14	F	c.7081C > T	p.Gln2361X
					2	F	c.6409G > T	p.Glu2137X
					31	F	c.6190_6212dup	p. Pro2071AsnfsX4
					41	F	c.6428T > C	p.Leu2148X
					20	M	c.6428T > C	p.Leu2148X
					46	F	c.6428T > C	p.Leu2148X
5	Descartes, M et al. [22]	2014	USA	1	47	F	c.6933delT	p.His2212Glnfs9
6	Han, M et al. [23].	2015	Korea	2	3	M	c.6854delA	p.Q2285Rfs2294
					32	F		
7	Deprouw, C et al. [24]	2015	France	1	36	F	Q2223X C > T	Not Reported
8	Battelino, N et al. [25]	2016	Slovenia	1	8	M	c.6909dup	p.lle2304Hisfs9
9	Giovani, A et al. [26]	2016	Italy	2	33	F	c.6667C > T	p.Gln2223Ter
					5	M		
10	Sakka, S et al. [27]	2017	England	4	15	F	c.6902T.A	p.Leu2301
					6	M	c.6662-6663delTG	p.Val2221GlufsX22
					15	M	c.6787C.T	p.Gln2263
					17	F	c.6724_6725delAG	p.Ser2242
11	Lee, J et al. [28].	2018	Korea	1	10	F	c.6787C > T	p.Gln2263
12	Swan, L et al. [29]	2018	Australia	1	NA	M	c.7066G > A	p.Ala2317Tr
13	Midro, A et al. [30]	2018	Poland	1	9	M	c.6424-6427delTCTG	p.Ser2142ArgfsX4
14	Pittaway J et al. [31]	2018	England	5	6	F	c.7198C > T	p.Arg2400X
					8 24	F	Not Reported	p.Pro2149Argfs 2×
15	This study	2020	Pakistan	1	37	M	c.6426_6427insTT	p.Glu2143Leufs*5

At present, definitive or effective pharmacological treatment for HCS is not available. The hallmark of the disease, however, is acroosteolysis and osteoporosis and, even though the exact molecular mechanism of bone loss is not yet known, bisphosphonate therapy is essentially empirical. According to the current knowledge that the main mechanism of bone disease is the activation of osteoclasts, through RANK activation, antiresorptive rather than anabolic treatment seems to be more appropriate; thus, most cases reported have been administered antiresorptive medications. As shown by our review, bisphosphonate therapy (Zoledronate, Pamidronate, and Alendronate), alone or as part of a treatment regimen, has been consistently utilized to counteract the progression of osteoporosis associated with the disease. Teriparatide and Denosumab have also been utilized for the skeletal manifestations of the syndrome. From what we were able to determine in our review, these therapies may not offer as much therapeutic benefit as that of Bisphosphonates (Table 5). This difference may likely be attributed to the variation in dosing regimens, as well as a lack of adequate data rather than simply being a measure of drug efficacy. Furthermore, our review also found differences between the therapeutic benefit of different Bisphosphonates. These results may, however, also be attributed to different scan intervals, dosing regimens, and age of the patients. As such, it is critical that further trials are done to determine the ideal symptomatic therapy for this disease.

**Table 5:** Treatment response of patients with HCS to various available anti-osteoporotic drugs reported in literature.

Class of Drugs	Ref No.	Treatment Regimen	Scan Interval (Years)	Lumbar Spine T/Z Score	
				Before Treatment	After Treatment
PTH Analogue	5	Teriparatide	Not Reported	−4.3	−3.0
Monoclonal Antibody	9	Denosumab	Not Reported	−4.2	Not Reported
Bisphosphonates	9	Zoledronic Acid	5	−4.1	−0.7
	10	Pamidronate	2	−3.1	−2.6
	12	Zoledronate	3	−3.4	−2.4
		Pamidronate Alendronate	6	−1.9	0.1
		Zoledronate	1½	−0.8	−0.7
		Zoledronate	2½	−2.1	−1.7
	16	Pamidronate	6	−3.1	−1.3
		Pamidronate Zoledronate	1½	−1.7	−0.3
			2½	−2.9	−2.5
		Pamidronate	3	−4.4	−4.5
		Alendronate	6	−3.0	−4.5
		Pamidronate	8½	−3.6	−3.0

In HCS, mutations in the terminal exon of NOTCH2 lead to the creation of a stop codon upstream of the PEST domain, responsible for ubiquitination and degradation of Notch, leading to a persistence of NOTCH2 signaling [1,4]. Hence NOTCH2 itself could be a future target for management of the disease, which would counteract the underlying pathophysiology of the disorder as opposed to the current symptomatic treatment available. Experimental modalities to control Notch signaling, including the use of antibodies to the Notch extracellular domain or its ligands and the use of cell membrane-permeable peptides that interfere with the formation of the Notch transcriptional complex [15,16] could be considered as future experimental therapies for HCS. These approaches, however, may result in severe unwanted events [17]. For example, impaired Notch signaling has resulted in gastrointestinal toxicity and vascular tumors in experimental animals [17]. Therefore, further controlled trials are warranted to ensure the successful implementation of a regimen better suited for the management of this disease. Moreover, the prognosis of HCS further worsens when basilar invagination causes neurologic complications, or thoracic deformities that might lead to respiratory restriction. Due to the low prevalence and the lack of quality of life information available about this syndrome, it is difficult to assess the years of healthy host life.

## 4 Conclusion

HCS due to NOTCH2 defect is an important differential diagnosis to consider in cases with acroosteolysis, osteoporosis, and multiple craniofacial anomalies. Gene testing can facilitate in reaching the correct diagnosis. The goal of treatment is to reduce the associated symptoms and to prevent osteoporotic fractures. To the best of our knowledge, this is the first case of Hajdu Cheney Syndrome to date which has been diagnosed and reported from Pakistan. This is not necessarily indicative of a decreased prevalence, however, but instead of a much larger issue regarding unavailability of the diagnostic tests in the country, requiring out sourcing to foreign labs, adding to the cost that has to borne by the family. Therefore, coupled with the rarity of the disease itself and the vast phenotypic spectrum with which it presents, the disorder can remain undiagnosed for years, especially in developing countries, as clinical suspicion is not enough to determine diagnosis. The resultant delay in treatment and management can lead to an overall increase in morbidity. In light of this, it is imperative that proper treatment and management strategies are devised alongside a structured diagnostic criterion as well as increased availability of molecular testing for rare diseases in Pakistan.

## Ethical approval

N/A.

## Sources of funding

None.

## Author contribution

SA, AA, SA and MOK performed the literature search, data collection and involved in the write-up of the work in the first draft. AHK conceived the idea, performed literature search, write-up and coordinated the writing of the paper and reviewed the final draft. SK was involved in the clinical and laboratory workup and critical revision of the article for the intellectual content. All authors have reviewed the final draft and agreed upon.

## Trial registry number

Not applicable.

## Guarantor

Dr Aysha Habib Khan.

Professor.

Section of Clinical Chemistry, Department of Pathology & Laboratory Medicine, the Aga Khan University Karachi, Pakistan.

Email: aysha.habib@aku.edu.

Phone:92-213-4861927.

## Consent

Written informed consent was obtained from the patient for publication of this case report. A copy of the written consent is available for review by the Editor-in-Chief of this journal on request.

## Provenance and peer review

Not commissioned, externally peer-reviewed.

## Declaration of competing interest

None.
